# Bisphenol a Exposure and Kidney Diseases: Systematic Review, Meta-Analysis, and NHANES 03–16 Study

**DOI:** 10.3390/biom11071046

**Published:** 2021-07-16

**Authors:** Rafael Moreno-Gómez-Toledano, María I. Arenas, Esperanza Vélez-Vélez, Elisabeth Coll, Borja Quiroga, Jordi Bover, Ricardo J. Bosch

**Affiliations:** 1 Universidad de Alcalá, Laboratory of Renal Physiology and Experimental Nephrology, Department of Biological Systems/Physiology, 28871 Alcalá de Henares, Spain; ricardoj.bosch@gmail.com; 2 Universidad de Alcalá, Department of Biomedicine and Biotechnology, 28871 Alcalá de Henares, Spain; misabel.arenas@uah.es; 3 Fundación Jiménez Díaz School of Nursing, Jiménez Díaz Foundation, Autonomous University of Madrid, 28040 Madrid, Spain; evelez@fjd.es; 4 Nephrology Service, Fundació Puigvert, 08025 Barcelona, Spain; ecoll@fundacio-puigvert.es; 5 Nephrology Service, La Princesa Universitary Hospital, 28806 Madrid, Spain; borjaqg@gmail.com; 6 Nephrology Service, Germans Trias i Pujol Hospital, Universitat Autònoma de Barcelona, 08916 Badalona, Spain; jordicatalonio@yahoo.es

**Keywords:** bisphenol A, kidney, systematic review, meta-analysis

## Abstract

Bisphenol A (BPA) is a compound that is especially widespread in most commonly used objects due to its multiple uses in the plastic industry. However, several data support the need to restrict its use. In recent years, new implications of BPA on the renal system have been discovered, which denotes the need to expand studies in patients. To this end, a systematic review and a meta-analysis was performed to explore existing literature that examines the BPA-kidney disease paradigm and to determine what and how future studies will need to be carried out. Our systematic review revealed that only few relevant publications have focused on the problem. However, the subsequent meta-analysis revealed that high blood concentrations of BPA could be a factor in developing kidney disease, at least in people with previous pathologies such as diabetes or hypertension. Furthermore, BPA could also represent a risk factor in healthy people whose urinary excretion is higher. Finally, the data analyzed from the NHANES 03-16 cohort provided new evidence on the possible involvement of BPA in kidney disease. Therefore, our results underline the need to carry out a thorough and methodologically homogeneous study, delving into the relationship between urinary and blood BPA, glomerular filtration rate, and urine albumin-to-creatinine ratio, preferably in population groups at risk, and subsequently in the general population, to solve this relevant conundrum with critical potential implications in Public Health.

## 1. Introduction

Bisphenol A (BPA) is a phenolic compound widely distributed in many everyday objects due to its multiple uses in the plastic industry [1]. Fundamentally used in the manufacture of polycarbonates and epoxy resins [2], BPA can be found in food and beverage containers, bottles and cans, as well as capsule coffee makers, tap water pipes, toys, electronic devices, and even in the composites used in dental restorations [3,4,5]. Increasing concerns over BPA as an endocrine-disrupting chemical and its possible effects on human health have prompted BPA removal from consumer products, often labeled “BPA-free”.

Currently, the European Food Safety Authority (EFSA) has delimited a tolerable daily intake (TDI) up to 4 µg/kg body weight/day, based on studies by Tyl et al. (2008) on the ability of BPA to affect the kidneys [6,7]. However, only a small percentage of publications study the BPA-kidney paradigm [8,9,10,11,12,13], compared to other organs and systems such as the reproductive or endocrine systems [14,15,16,17,18,19,20]. In this sense, previous studies developed by our team determined that mouse podocytes (cells that are part of the glomerular filtration barrier) treated with BPA (10 and 100 nM) underwent apoptosis followed by podocytopenia, albuminuria [12], and hypertension. Interestingly, when treating cultured human podocytes at the same doses of BPA, we observed a podocytopathy characterized by impaired cell adhesion due to alteration in the expression of the podocyte’s adhesion and structural proteins [13]. Furthermore, epidemiological studies in humans have found interesting relationships between BPA concentration in blood or urine and the risk of developing kidney disease [21,22,23,24].

In parallel, other works by our team determined that BPA could influence the development of arterial hypertension, a disease closely related to kidney pathologies. When mice were treated with BPA in drinking water (20–25 mg/kg/day), significant increases in blood pressure were observed even at low doses [25]. The results are consistent with epidemiological studies, such as Shankar et al. [26], in which they observed a positive relationship between urinary BPA excretion and hypertension, independent of other factors. Furthermore, Bae et al. [27] observed that people who consumed canned drinks vs. glass bottles had up to 1600% urinary BPA and a higher variation in systolic pressure.

These data suggest that BPA could play a role in the pathogenesis and progression of renal diseases. Consequently, it is essential to study the possible implications of BPA in the renal system of the healthy population and patients with kidney disease. A preliminary search was conducted to verify whether there is any systematic review or meta-analysis in the academic literature, using the keywords “bisphenol AND (systematic review OR meta-analysis) AND (renal OR kidney)”.

The preliminary analysis only showed one relevant article [28]. The publication only studied the potential effect of BPA on the pediatric population, which means that the approach of the present work is novel in the academic literature. Therefore, a thorough review was carried out using the methodology of systematic reviews to analyze potentially deleterious renal effects of BPA and describe potential sources of bias. Next, the available data were analyzed to carry out a meta-analysis that combined all possible studies. Finally, in the absence of publications that study the data set of the NHANES 2003–2016 cohort, a brief analysis was carried out that could provide new evidence on the BPA-kidney paradigm.

## 2. Materials and Methods

### 2.1. Study Selection

Studies that reported the association between high exposure to BPA in humans (in urine or blood) and risk of kidney disease were eligible for inclusion. Due to the small number of existing publications, all available population groups and age ranges were studied. To carry out the meta-analysis, all those studies with an available odds ratio (OR) and confidence interval (CI) or, at least, with sufficient data allowing calculation of the latter parameters were selected. All publications with any degree of relationship between BPA and kidney disease were selected.

### 2.2. Information Sources and Search Strategy

This search was carried out during July 2021 using the reference academic search engines PubMed (pubmed.ncbi.nlm.nih.gov, accessed on 5 July 2021) and Web of Science (apps.webofknowledge.com, accessed on 5 July 2021). To optimize the search strategy, we combined the terms “Bisphenol AND (urine OR urinary OR serum OR kidney OR renal) AND (albuminuria OR microalbuminuria OR nephropathy OR glomerular OR interstitial OR vascular OR CKD)”, without year restriction.

After eliminating the repeated articles, using Mendeley (Mendeley Desktop, V. 1.19.4, Mendeley Ltd., Elsevier, London, United Kingdom) manager and organizer, we evaluated the resulting academic papers by title/abstract (Discarding all publications that do not fit the review context, such as in vitro research, animal models, or the study of compounds other than BPA, such as BADGE or BPS. Subsequently, after conducting the first preliminary study, each of the academic articles selected was evaluated, with the following eligibility criteria:

- Original data (excluding bibliographic reviews) accepted and published.
- Studies in human populations (adults or children).
- Quantification of BPA in urine or serum and its correlation with any predisposition or susceptibility marker of kidney damage or disease.
- Studies published in English.

Two independent reviewers (RMGT and RJB), whose decisions in each of the bibliographic search and evaluation steps were determined by consensus, evaluated all the publications.

### 2.3. Data Collection and Quality Assessment

Once the publications of interest were selected, they were classified according to the study variable. Three studied BPA in the blood, while the rest analyzed it in the urine. Simultaneously, other important parameters for determining kidney damage were also considered since two articles explored the concept of “low-grade albuminuria,” while others investigated the estimated glomerular filtration rate (eGFR). Notably, the studies that evaluated blood BPA were carried out on diagnosed or developing pathologies, while the urinary studies mainly presented data from the healthy population.

### 2.4. Analytical Method

At the statistical level, the objective was to perform the inverse analysis of variance to unify the studies related to each other, introduced in a random-effects model. The steps followed for the analysis of each subgroup are detailed below.

#### 2.4.1. BPA in Blood

In order to calculate the OR, we analyzed the following articles: Krieter et al. [24], Hu et al. [29], Hu et al. [22], and Shen et al. [30]. In the case of Krieter and Shen, their studies show the mean BPA in plasma depending on kidney damage. In contrast, in the case of Hu’s, it shows the OR of the probability of developing kidney damage in the presence of high concentrations of serum BPA in patients with diabetes [29] and hypertension [22]. As there were already 2 ORs, the first step that has been carried out has been to calculate it for Krieter et al. [24] and Shen et al. [30].

**Figure 1:**
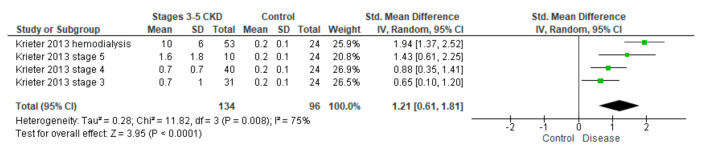
Mean difference analysis using the RevMan program calculates a value to use in the inverse variance analysis [24].

Review Manager (RevMan 5.3, Cochrane, London, United Kingdom)was used to calculate the standardized mean difference comparing the control group with the different concentrations of BPA in stages 3 to 5 of chronic kidney disease (CKD) (in Hu et al. [22,29], the patient was considered to have kidney disease when he reaches stage 3, that is when clearance is below 60 mL/min/1.73 m^2^). The four subgroups analyzed correspond to stages 3, 4, 5, and stage 5 undergoing hemodialysis (Figure 1).

Once the standardized mean difference has been obtained, the OR can be obtained by applying the formula [31]:

Standardized mean difference = (√3/π) × ln Odds Ratio,

Since the standard error is also required, the following formulas were applied to the CI obtained [31]:

Standard deviation (SD) = √N × (upper limit−lower limit)/3.92,

Standard error (SE) = Standard deviation/√N

**Figure 2:**
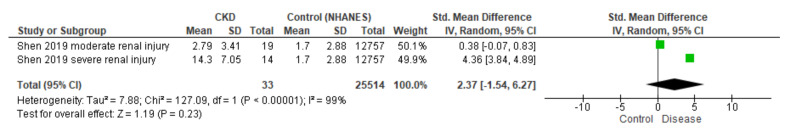
Mean difference analysis using the RevMan program calculates a value to use in the inverse variance analysis [30].

In the case of Shen et al. [30], patients with moderate renal injury [30 ≤ eGFR < 60 mL/min/1.73 m^2^], and severe renal injury [eGFR < 30 mL/min/1.73 m^2^] were included (Figure 2). In this case, as a control BPA value could not be found, the average of the NHANES cohort was used (see Table 1).

#### 2.4.2. BPA in Urine

Several analyses were carried out, depending on the parameter related to kidney damage. Firstly, two publications studied the concept of low-grade albuminuria; it is understood as an increase in urinary albumin excretion but consistently below the limit considered pathological (30 mg/g creatinine). In this regard, Li et al. [23] and Trasande et al. [21] considered that when studying the “healthy” population, those individuals who were in quartile 4 of urinary BPA have a higher probability of presenting with a higher concentration of albuminuria. Li et al. [23] provides an OR that can be used, but Trasande et al. [21] shows a standardized mean difference, so the formulas previously described must be applied.

On the other hand, in the following two studies conducted by Malits et al. [22] and Kang et al. [32], Lee et al. [33], and Kang et al. [34] the association between BPA and urine albumin-to-creatinine ratio (ACR) were studied. From the work data, an OR could be obtained with CI in all cases, so the logarithms and the corresponding standard errors were calculated.

**Figure 3:**
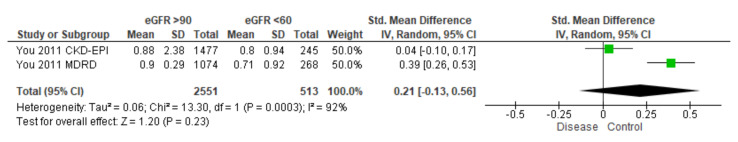
Difference of means of the groups elaborated from the MDRD and CKD-EPI formulas for the estimation of creatinine clearance [35].

Secondly, the relationship between BPA and the eGFR has also been studied in the studies by You et al. [35], Malits et al. [36], Lee et al. [33], Jacobson et al. [37], and Kang et al. [34]. Malits et al. [36], Lee et al. [33], Jacobson et al. [37], and Kang et al. [34] offer enough data, but in the work of You et al. [35], OR must be calculated from the standard mean difference of its two study groups. They use the Modification of Diet in Renal Disease (MDRD-4) and the “Chronic Kidney Disease Epidemiology Collaboration” (CKD-EPI) equations to estimate GFR (Figure 3).

Next, the OR with its 95% CI was used to calculate the estimated pooled effect. Heterogeneity between studies was calculated by applying the Chi^2^ and I^2^ tests. The I^2^ statistic was calculated as a percentage, and they were interpreted as low, medium, or high heterogeneity, reaching 25, 50, and 75%, respectively [38]. The *p*-value < 0.05 was considered statistically significant for all the analyses performed. The statistical program used to carry out the analyses was Review Manager (RevMan 5.3).

### 2.5. Study of the NHANES 03-16 Cohort

After studying the academic literature, the absence of studies using all the BPA values in the NHANES 2003–2016 cohort was observed, and the analysis of the subpopulations classified as kidney disease during the patient evaluation process undiagnosed kidney disease whose eGFRs are abnormal. Therefore, this section aims to unify in a single database all the American populations’ urinary BPA measurements studied from 2003 to 2016 (data currently available) to carry out the statistical study on the relationship between BPA and kidneys with the largest sample size described to date.

Patient files containing elements like urinary bisphenol, albumin and creatinine levels, plasma creatinine, sex, weight, age, kidney diseases, or dialysis patient were first collected to correlate urinary BPA with renal function. All files can be found in Xps Transport File Format (.XPT) and downloaded from “https://wwwn.cdc.gov/ (accessed on 16 July 2021)” Once the data had been extracted, unified, and classified according to each patient’s numerical code, a table was obtained with 72,697 people (numerical code 21,005 to 93,702). The adults (age > 18 years) were selected, and all those in whom urinary BPA was not quantified were eliminated, obtaining a sample size of 12,757 people.

Spot urine samples are unable to determine urinary volume or albuminuria excreted within 24 h. Therefore, the ACR (mg of albumin/g of creatinine) was used to estimate albumin excretion. Similarly, the excretion of BPA to urinary creatinine (µg BPA/g creatinine) was also adjusted for the same reason. The MDRD-4 and the CKD-EPI equations (the most widely used formulas) are used to calculate the eGFR [39,40,41].

NHANES data were analyzed using the GraphPad Prism 7.0 software (GraphPad Software Inc., San Diego, CA, USA). The data were analyzed using the D’Agostino & Pearson and Kolmogorov–Smirnov normality tests. As they did not follow a normal distribution, the data are expressed as median (95% CI) in the tables and the graphs. The Kruskal–Wallis test followed by Dunn´s multiple comparisons test or Mann–Whitney test was performed. Differences were considered statistically significant at *p* < 0.05.

## 3. Results

### 3.1. Selection of Academic Articles

**Figure 4:**
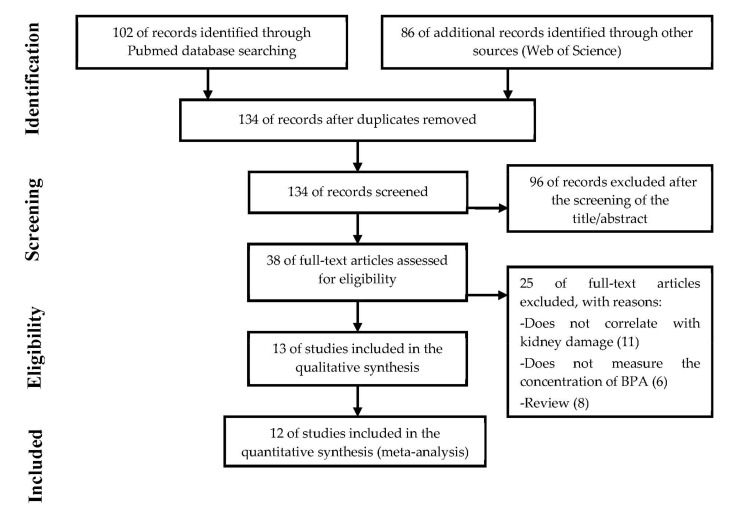
Schematic representation of the methodology used based on The PRISMA Statement [44].

The initial search identified 102 articles in PubMed and 86 in Web of Science. After exporting all references to the Mendeley application and eliminating duplicates, 134 items were placed. The selection process was carried out based on the information contained in the title/abstract (in vitro studies, in vivo, reviews/comments). Publications related to quantification methodologies were eliminated, as well as those that studied organs/compounds different from kidney/BPA. After the first screening, 38 articles were identified, and the full texts were reviewed. Of these, twelve were included in the systematic review (Figure 4).

### 3.2. Study Characteristics

We will proceed to briefly describe the most interesting aspects of the academic publications used in the meta-analysis (for more information, see Supplementary Table S1). Firstly, the studies evaluating BPA in blood samples correspond to Krieter et al. [24], Hu et al. [22,29], Nie [42], and Shen et al. [30]. In the first one [24], the study group consisted in 152 patients with different CKD stages and 24 healthy subjects. The eGFR was calculated using the MDRD equation and compared with the BPA concentrations in plasma (ng/mL). An inverse relationship between plasma BPA and renal function (GFR) was observed. The highest plasma concentrations were present in patients with the lowest GFR (stage 5). In Hu et al. [29], they studied the relationship between serum BPA and the predisposition to develop CKD in 121 patients with type 2 diabetes over six years. Patients with high serum BPA concentrations had almost seven times the risk of developing CKD than patients with lower BPA concentrations (OR 6.65). In the 2016 publication [22], after studying 302 patients with primary hypertension, they determined that those with higher concentrations of serum BPA were five times as likely (OR 4.79) to develop stage 3 CKD or higher. In both studies, they observed an interesting negative correlation between serum BPA and estimated glomerular filtration rate (β = −0.371 and −0.132, in the 2015 and 2016 studies, respectively). Finally, in the work of Shen et al. [30], a negative correlation between serum BPA and eGFR was also observed, as well as a significant elevation of plasma BPA in those patients with severe CKD.

Secondly, the academic publications that studied BPA in urine can be subdivided according to the renal parameter studied. The first group of interest corresponds to the two publications that use the concept of low-grade albuminuria. When classifying the “healthy” population (ACR below 30 mg/g) into four quartiles, it has been observed that those subjects who were in quartile four could have a greater predisposition to specific pathologies, such as cardiovascular disease [43]. Within this group were the works of Li et al. [23] and Trasande et al. [21]. In the first of them, they selected from among 3455 Chinese adults, all with ACR lower than 30 mg/g and considered those located in quartile four as presenting low-grade albuminuria. Finally, multivariate stepwise linear regression analysis determined that urinary BPA concentration was an independent predisposing factor to low-grade albuminuria. The other publication studied 710 American children from the American NHANES cohort (National Health and Nutrition Examination Survey). Children were categorized according to their urinary BPA concentration into four quartiles, observing that those in the 4th quartile had a significantly higher ACR than the individuals in the first quartile. They determined a 0.28 mg/g ACR increase for each log unit increase in urinary BPA after performing multinomial regression models.

Within the other group can be found the works of You et al. [35], Malits et al. [36], Kang et al. [32], Lee et al. [33], Jacobson et al. [37], and Kang et al. [34]. The first of them [35], studied kidney function by calculating the eGFR using the MDRD and CKD-EPI equations. They subdivided the population into three categories for each equation: ≥90 mL/min/1.73 m^2^, normal kidney function; 60–90 mL/min/1.73 m^2^, mild alteration; ≤60 mL/min/1.73 m^2^, CKD. They performed regression models and adjusted for factors related to health, socioeconomic factors, and urinary creatinine. The results showed a positive and statistically significant relationship between urinary BPA excretion and the eGFR so that pathological situations, those with a lower GFR, presented lower concentrations of urinary BPA. The second publication compared a cohort of children with CKD with a general cohort (NHANES), determining that children with CKD have urinary BPA concentrations lower than those observed in healthy children. However, multivariate linear regression (corrected for health and social factors) showed a negative relationship between urinary BPA and eGFR.

Kang et al. [32] shows a statistically significant negative relationship between creatinine-corrected BPA and ACR in different regression models when studying a population of 441 Korean adult women (adjusted for age, region, education, and diabetic, obesity and cardiovascular factors). Lee et al. [33] determined a statistically significant negative relationship between BPA and eGFR in the general Korean population. Jacobson et al. [37] observed, in children with kidney disease, a negative relationship between BPA and eGFR (linear mixed-effects models). Interestingly, they also determined a positive and statistically significant relationship between urinary BPA and kidney damage markers, such as NGAL and KIM-1. The work of Kang et al. [34] also determined a strong negative correlation between BPA and eGFR after using a procedure of covariate-adjusted creatinine standardization. Finally, the last publication should be mentioned that did not provide usable data in the urine meta-analysis but is relevant in the BPA-kidney paradigm, is Jain [45]. In this study, it was observed that the association between urinary BPA and ACR could vary depending on kidney function, determining positive or negative relationships depending on the stage of kidney disease.

### 3.3. Meta-Analysis

As previously mentioned, the BPA-kidney damage paradigm comprises a small number of quite heterogeneous but potentially significant publications.

#### 3.3.1. BPA in Blood

**Figure 5:**
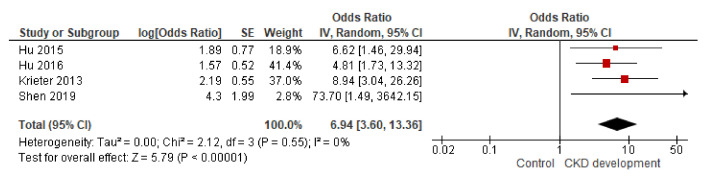
Analysis of the inverse variance of the four publications that study BPA in blood and kidney disease [22,24,29,30].

Once all the ORs and their respective standard errors were obtained, the inverse variance method was calculated. As shown in Figure 5 [OR 6.94, 95% CI 3.60–13.36], higher concentrations of BPA were related to a greater risk of developing kidney damage. The heterogeneity tests demonstrated that the data were excellently homogeneous.

#### 3.3.2. BPA in Urine

**Figure 6:**
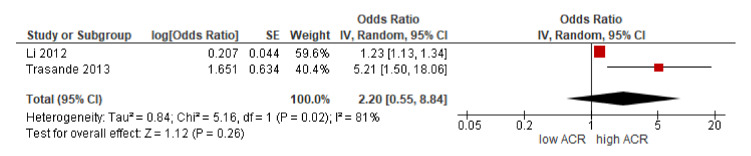
Analysis of the inverse of variance of the two publications that study BPA in urine and low-grade albuminuria [21,23]. ACR: urine albumin-to-creatinine ratio.

The unified works by Li et al. [23] and Trasande et al. [21], based on patients with low-grade albuminuria, showed high heterogeneity. The OR was positive, but the CI was vast, with its range including values below 1 (Figure 6). There was a combined probability of 2.2-fold for developing low-grade albuminuria with high urinary concentrations of BPA.

**Figure 7:**
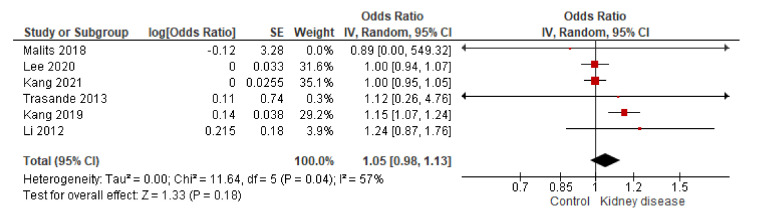
Analysis of the inverse variance of the publications that study BPA in urine and ACR [21,32,33,34,36].

In the study between urinary BPA and ACR, the combined effect is slightly positive. There are large SEs in some studies, so the weight of the study falls on three works [33,34]. The combined results show a slight positive relationship between urinary BPA and albuminuria (Figure 7).

**Figure 8:**
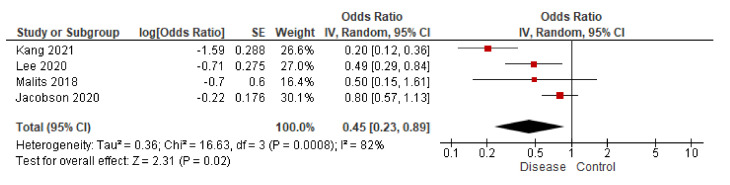
Study of the inverse variance in publications that determine the relationship between BPA and eGFR [33,34,36,37].

Finally, in studying the relationship between BPA and eGFR, four studies show a negative relationship between both parameters in their respective multiple linear regression studies (corrected with demographic, social, and health parameters). Only the work of You et al. [35] showed an inverse result (remember that the OR was calculated through the difference of means). Figure 8 shows the combined effect of the four studies with the same methodology, evidencing the negative relationship observed in all publications. In this case, the reduction in eGFR is related to a loss of kidney function. By including the work of You et al. [35], a combined OR (95% CI) of 0.59 (0.30, 1.16) is obtained. In any case, it could be considered that there is sufficient evidence to suggest that BPA could influence the development of CKD, reducing eGFR.

### 3.4. NHANES 03-16 Cohort

As can be seen in Table 1, more than 12,000 data from adult individuals were analyzed. The proportion of men and women was practically the same. The urinary BPA concentration was relatively low and consistent with the data observed in the general population [46]. Men had significantly higher BPA values per mL of urine but significantly lower BPA corrected for creatinine. On the other hand, men presented significantly lower values of eGFR and ACR but higher values of serum creatinine and albumin.

**Figure 9:**
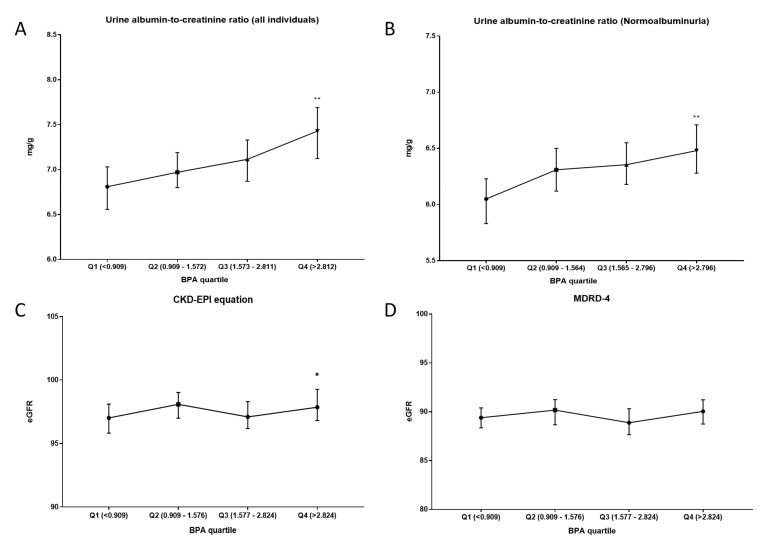
Schematic representation of the results obtained in the parameters related to renal function according to the BPA quartile. (**A**) Urine albumin-to-creatinine ratio (mg/g) in all individuals of the cohort. (**B**) Urine albumin-to-creatinine ratio (mg/g) only in normoalbuminuric individuals (<30 mg/g). (**C**) CKD-EPI equation for eGFR. (**D**) MDRD-4 equation for eGFR. All results were expressed as median (95% CI). Kruskal–Wallis test followed by Dunn´s multiple comparisons test or Mann–Whitney test was performed. * *p*-value < 0.05; ** *p*-value < 0.01.

By analyzing the data based on BPA/creatinine quartile, a significant increase in the ACR was manifested when comparing the group with higher levels of urinary BPA (Q4) with those with the lowest amount (Q1) (Figure 9A). Similarly, when analyzing all people with a ratio below 30 mg/g, the same trend was observed (Figure 9B); people belonging to Q4 showed a significant increase in the ratio compared to those in Q1. The estimated clearance study using the CKD-EPI and MDRD-4 equations can observe significant differences between Q4 and Q1 only in the CKD-EPI equation (Figure 9C,D).

**Figure 10:**
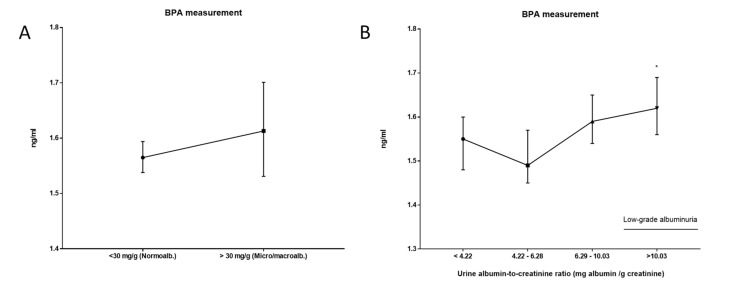
Schematic representation of the results obtained in the urinary BPA according to the urine albumin-to-creatinine ratio. (**A**) < 30 mg/mL (normoalbuminuria) vs. >30 mg/Dl (micro/macroalbuminuria). (**B**) Normoalbuminuria. The population was classified into four quartiles. Those in Q4 are considered to have low-grade albuminuria. All results were expressed as median (95% CI). Kruskal–Wallis test followed by Dunn´s multiple comparisons test was performed. * *p*-value < 0.05.

When analyzing urinary BPA concentrations as a function of ACR, no differences were observed between individuals with non-pathological values (<30 mg/g, normoalbuminuria) vs. individuals with pathological values (>30 mg/g, micro/macroalbuminuria) (Figure 10A). However, when classifying individuals with normoalbuminuria into four quartiles, those in quartile 4 (theoretically they would present low-grade albuminuria [21,23]) had a higher concentration of urinary BPA than individuals in quartile 1 (Figure 10B).

Among the individuals in the cohort were patients with kidney disease and some on dialysis. When studying BPA corrected for creatinine, significant increases were observed in patients with kidney disease vs. healthy subjects, and patients undergoing dialysis vs. patients without treatment (Table 2).

## 4. Discussion

Firstly, the systematic review shows an evident scarcity of publications that study the BPA-kidney paradigm in humans. However, it is interesting to note that the limitations on exposure to BPA proposed by the EFSA base their model on animal models that study how this compound affects the kidney [6,7].

Secondly, the meta-analysis shows that the high concentration of BPA in the blood could be a warning sign for kidney disease. Increasing the concentration of a compound in the blood due to the kidney’s reduced filtering capacity is logical from a functional point of view. If we only look at the work of Krieter et al. [24] and Shen et al. [30], it could be deduced that this increase is a logical consequence of the kidney’s inability to perform its work naturally. However, the two studies by Hu et al. [22,29], both longitudinal, analyze their study group over time, which gives greater strength to the hypothesis that elevated BPA in the blood could be a predisposing factor to kidney damage, at least within patients with previously diagnosed pathologies such as hypertension or diabetes. Furthermore, these authors identified high levels of serum BPA as a predictor of CKD in two of the most common renal conditions, such as hypertension and diabetic nephropathy.

On the other hand, a high urinary BPA concentration could be related to an increase in the excretion of albuminuria in the healthy population. Perhaps it would be possible that in the healthy population, whose filtering capacity is standard, the increase in excreted BPA could be related to a greater predisposition to increased albuminuria, which could be an early indication of kidney damage. However, some publications suggest that BPA could bind to proteins [47], which would be a consequence of increased albuminuria and not a cause. In the general study of the ACR, a positive relationship was observed. The results’ limitations show the need to deepen these aspects with larger sample groups or focus on specific pathologies and carry out new longitudinal studies. In this sense, it would be interesting to observe BPA’s urinary excretion in patients in advanced stages of CKD.

BPA is a compound whose metabolism is fundamentally characterized by phase II reactions, biochemical mechanisms capable of modifying its structure to facilitate its excretion [48]. This detoxification capacity can be altered in diseases such as obesity or diabetes [49]. Glucuronidation is the majority reaction, mediated by uridine diphosphate glucuronosyltransferase (UGT) [48,50]. Pharmacokinetic studies have determined that BPA is excreted exclusively through the urine in non-human primates and humans [51,52,53]. For this reason, the correlation between pathologies and urinary BPA is a powerful tool since the actual exposure to BPA is theoretically taken into account.

Within the study’s limitations, it must be considered that the blood studies were carried out on specific population groups, such as patients with kidney disease, type 2 diabetes, and hypertension. In contrast, the urinary studies were carried out in the general population, except that of Malits et al. [36] and Jacobson et al. [37], where they employed a group of children with CKD.

The analysis of eGFR and blood BPA suggests that higher concentrations of BPA present a greater probability of suffering kidney damage (or lower eGFR). However, in the works of You et al. [35] and Malits et al. [36], a lower concentration of urinary BPA is observed in patients with kidney disease. This relationship changes after statistical analysis, which is corrected with social, anthropometric, and health factors, obtaining a negative relationship. It is coherent to think that a damaged kidney with a limited glomerular filtration capacity will not efficiently eliminate BPA from the body, reduce its excretion, and increase its concentration in the systemic circulation. However, a statistically corrected analysis shows that urinary BPA can also be significantly correlated with eGFR. The absolute value of urinary BPA may be affected by parameters such as urinary volume or creatinine, but the statistical relationship is quite strong.

Kidney disease is closely related to diseases such as hypertension. In this sense, it has been observed that BPA could be a predisposing factor for the development of hypertension in animal models [25] or epidemiological studies [26,27]. Recently, in 2021, a study has been published unifying all the available data on BPA and cardiovascular disease, obtaining an OR of 1.13 (95% CI, 1.03–1.23) [54].

The main conclusion that can be drawn from the meta-analysis is the need to carry out a complete study, which delves into the relationship between urinary and plasma BPA, glomerular filtration rate, and albuminuria, both in population risk groups (in patients with diabetes or hypertension) and in the general population. This study must be placing a particular emphasis on the particularities related to the ACR and glomerular filtration (which could be calculated with the main formulas currently used in clinical practice, which are the MDRD-4 and CKD-EPI [55]). 

Finally, the data analyzed from the NHANES cohort show new evidence that reinforces the hypothesis that BPA acts as a factor involved in kidney damage. It is interesting to highlight the importance of creatinine correction in studying urinary metabolites, such as BPA. Thus, a significant increase in urinary BPA has been observed in men, expressed in µg/mL, while women presented higher BPA values corrected for creatinine. In the absence of the absolute volume of urine excreted in 24 h, correction for creatinine allows a better estimation of urinary metabolites, taking into account the renal filtration capacity.

Regarding the analysis of the population, classified according to urinary BPA, evident and significant differences have been observed between people with lower and higher concentrations of urinary BPA (Q1–Q4). In the study of the complete cohort and the study of the healthy population (normoalbuminuria), significant increases in the ACR between Q4 and Q1 have been observed. Similarly, significant increases in urinary BPA were observed in those individuals with low-grade albuminuria. This data is consistent with the results observed by Li et al. [23] and Trasande et al. [21] (Figure 6). On the other hand, only when the CKD-EPI formula was used, significant changes between Q4 and Q1 were observed. Since BPA is a metabolite that constantly enters the body through multiple sources and is constantly excreted in the urine due to its low half-life, it makes sense that with greater glomerular filtration, there is greater BPA excretion. This data is not of great relevance to demonstrate a causal relationship between the BPA-kidney paradigm; however, urinary BPA concentrations in patients with kidney disease in the cohort are of special interest. As observed in Table 2, the kidney disease patients had higher urinary BPA levels than the rest of the subjects in the cohort. Similarly, patients undergoing dialysis treatment had higher BPA concentrations than those who were not yet receiving treatment.

In short, the body of evidence in the present work takes a further step towards the relationship between BPA and the predisposition to develop kidney diseases. BPA may bind to proteins, limiting the correct analysis of parameters such as ACR, and it seems that the glomerular filtration capacity conditions its excretion rate. However, the joint analysis of the studies on BPA in blood, together with the urinary concentrations observed in the NHANES cohort patients, provides new evidence that supports this possible causal relationship. Although experimental data fully support a pathogenic role of BPA exposure on hypertension and renal disease, further epidemiological studies need to clarify this critical issue.

## 5. Conclusions

- Despite the importance of the BPA-kidney paradigm in the context of human exposure, few works explore the issue.
- In the study of blood BPA and kidney disease, solid evidence correlates high concentrations of BPA in the blood with a greater predisposition to develop kidney disease, at least under pathological conditions.
- In the study of the ACR and urinary BPA, a positive relationship was observed in healthy subjects. The same trend was observed in the NHANES cohort. Similarly, subjects with low-grade albuminuria showed a significant increase in urinary BPA.
- Despite inconsistencies observed in urinary BPA concentration from patients with kidney disease, statistical correlations with eGFR support an important relationship between BPA and glomerular filtration.
- The results, consistent with the experimental models, show interesting evidence that positions BPA as a possible environmental factor inducing kidney damage.

## Figures and Tables

**Table 1:** Summary of the adult population studied in NHANES 03-16. The data were analyzed using the D’Agostino & Pearson and Kolmogorov–Smirnov normality tests. Data were represented using the median (95% CI). For statistical analysis, the Mann–Whitney test was performed.

	N (%)	Age, Years	Urinary BPA, ng/mL	Urinary BPA, µg/g Creat.	MDRD-4 (eGFR)	CKD-EPI (eGFR)	Ratio alb./Creat.	Serum Creat., mg/dL	Serum Albumin, g/dL
Male	6238 (48.49%)	47 (46–48)	1.8 (1.7–1.9)	1.4 (1.37–1.44)	88.52 (87.92–89.12)	95.25 (94.55–96.05)	6.06 (5.9–6.21)	0.98 (0.97–0.99)	4.4 (4.4–4.4)
Female	6519 (51.10%)	46 (45–46)	1.6 (1.5–1.6)	1.75 (1.71–1.78)	90.75 (89.88–91.57)	99.94 (99.18–100.7)	7.98 (7.78–8.15)	0.73 (0.72–0.74)	4.2 (4.2–4.2)
Total	12757 (100%)	46 (46–47)	1.7 (1.6–1.7)	1.57 (1.55–1.6)	89.96 (88.97–90.21)	97.48 (96.99–98.09)	7.06 (6.95–7.18)	0.85 (0.84–0.85)	4.3 (4.3–4.3)
*p*-value		0.0642	<0.0001	<0.0001	<0.0001	<0.0001	<0.0001	<0.0001	<0.0001

**Table 2:** Urinary BPA/creatinine in diabetic and kidney patients vs. healthy population. Data were represented using the median (95% CI). For statistical analysis, Mann–Whitney test was performed.

Group	Urinary BPA, µg/g Creatinine	n	*p*-Value
Kidney disease	1.784 (1.5–2)	320	0.016
The rest of the cohort	1.563 (1.538–1.591)	11,572	
Dialysis treatment	2.9 (1.744–3.75)	24	0.031
Non-dialysis kidney patient	1.677 (1.458–1.942)	296	

## Data Availability

Not applicable.

## Supplementary Materials

The following are available online at https://www.mdpi.com/article/10.3390/biom11071046/s1, Table S1: Summary of the main characteristics of the papers included in the systematic review.
