# Supplementary material for: Bisphenol a Exposure and Kidney Diseases: Systematic Review, Meta-Analysis, and NHANES 03–16 Study
Source: Biomolecules. 2021 Jul 16;11(7):1046. doi: 10.3390/biom11071046 (PMC8301850; doi:10.3390/biom11071046)
Supplement: Supplementary file 1 [file biomolecules-11-01046-s001.zip › biomolecules-1272534-supplementary.pdf]

Table S1. Summary of the main characteristics of the papers included in the systematic review. Abbreviations: CKD, Chronic Kidney Disease; GM, Geometric Mean; CI, Confidence Interval; GSD, Geometric Standard Deviation); SD, Standard Deviation; IR, Interquartile Range; ITR, Intertertile Range; BPA e, BPA exposed; BPA non-e, BPA non-exposed; SRI, Severe Renal Injury; MRI, Moderate Renal Injury.

Study	Year	Country	Study design	Sample size	Sex	Age, Median (Range), years / Mean±SD	Exposure (ng/ml)	Exposure assessment	Study period (years)	Biologic fluid
You [1]	2011	USA	Cross-sectional	2573	Males and females	47.12 (>20 years)	2.04 (1.76, 2.36) – 2.46 (2.41, 2.50) GM (95%CI)	HPLC-MS after deconjugation	2003-2006	Urine
Li [2]	2012	China	Cross-sectional	3455	Males and females	60.8 ± 9.9	0.81 (0.48 - 1.44) Median (IR)	HPLC-MS after deconjugation	2008-2009	Urine
Trasande [3]	2013	USA	Cross sectional	710	Males and females	(6-11)<br>(12-19)	2.25 Mean<br>1.76 Mean	HPLC-MS after deconjugation	2009-2010	Urine
Krieter [4]	2013	Germany	Cross-sectional (Case-control)	152 CKD patients + 24 healthy	Males and females	69.7 ± 12.6	CKD stage 5 with dialysis 10 ± 6.6 Mean (SD)	ELISA	-----	Plasma
Hu [5]	2015	China	Longitudinal study	121 type 2 diabetes patients	Males and females	65.13 ± 10.57	0.40 (0.174, 1.40) Median (ITR)	ELISA	2008-2014	Serum
Hu [6]	2016	China	Longitudinal study	302 hypertensive patients	Males and females	65.29 ± 9.78	0.61 (0.26, 2.44) Median (ITR)	ELISA	2008-2014	Serum
Malits [7]	2018	USA	Cross sectional (case-control)	538 CKD patients	Males and females	10.77 ± 0.18 (1-17)	0.69 (0.61, 0.78) GM (95% CI)	HPLC-MS after deconjugation	2005-2008; 2009-2014	Urine
Kang [8]	2019	Korea	Cross-sectional	441	Female (before menopause)	34.77 (20-48)	0,5 median	HPLC-MS after deconjugation	2015-2016	Urine
Shen [9]	2019	China	Cross sectional	58 CKD,30 healthy	Males and females	63.12 ± 6.31 (BPA e)<br>60.45 ± 6.95 (BPA non-e)	SRI: 14.30 (8.93, 16.32)<br>MRI: 2.79 (1.01, 4.08) GM (95% CI)	HPLC-MS after deconjugation	-----	Serum
Lee [10]	2020	Korea	Cross sectional	1292	Males and females	(19 - >70)	1.31 (0.51, 2.72) Median (IR)	UPLC-MS/MS after deconjugation	2015-2017	Urine
Jacobson [11]	2020	USA and Canada	Cross sectional	618	Males and females	11 (7.6, 14.6) Median (IR)	0.59 (3.49) GM (GSD)	HPLC-MS after deconjugation	2005-2015	Urine
Kang [12]	2021	USA	Cross sectional	9008	Males and females	(20 – 79)	1.5 (0.7, 3) GM (IR)	HPLC-MS after deconjugation	2005 - 2016	Urine

References

1. You, L.; Zhu, X.; Shrubsole, M.J.; Fan, H.; Chen, J.; Dong, J.; Hao, C.-M.M.; Dai, Q. Renal function, bisphenol A, and alkylphenols: Results from the National Health and Nutrition Examination Survey (NHANES 2003-2006). *Environ. Health Perspect.* **2011**, *119*, 527–533, doi:10.1289/ehp.1002572.
2. Li, M.; Bi, Y.; Qi, L.; Wang, T.; Xu, M.; Huang, Y.; Xu, Y.; Chen, Y.; Lu, J.; Wang, W.; et al. Exposure to bisphenol A is associated with low-grade albuminuria in Chinese adults. *Kidney Int.* **2012**, *81*, 1131–1139, doi:10.1038/ki.2012.6.
3. Trasande, L.; Attina, T.M.; Trachtman, H. Bisphenol A exposure is associated with low-grade urinary albumin excretion in children of the United States. *Kidney Int.* **2013**, *83*, 741–748, doi:10.1038/ki.2012.422.
4. Krieter, D.H.; Canaud, B.; Lemke, H.-D.D.; Rodriguez, A.; Morgenroth, A.; von Appen, K.; Dragoun, G.-P.P.; Wanner, C. Bisphenol A in Chronic Kidney Disease. *Artif. Organs* **2013**, *37*, 283–290, doi:10.1111/j.1525-1594.2012.01556.x.
5. Hu, J.; Yang, S.; Wang, Y.; Goswami, R.; Peng, C.; Gao, R.; Zhou, H.; Zhang, Y.; Cheng, Q.; Zhen, Q.; et al. Serum bisphenol A and progression of type 2 diabetic nephropathy: a 6-year prospective study. *ACTA Diabetol.* **2015**, *52*, 1135–1141, doi:10.1007/s00592-015-0801-5.
6. Hu, J.; Wang, Y.; Xiang, X.; Peng, C.; Gao, R.; Goswami, R.; Zhou, H.; Zhang, Y.; Zhen, Q.; Cheng, Q.; et al. Serum bisphenol A as a predictor of chronic kidney disease progresion in primary hypertension: A 6-year prospective study. *J. Hypertens.* **2016**, *34*, 332–337, doi:10.1097/HJH.0000000000000780.
7. Malits, J.; Attina, T.M.; Karthikraj, R.; Kannan, K.; Naidu, M.; Furth, S.; Warady, B.A.; Vento, S.; Trachtman, H.; Trasande, L. Renal Function and exposure to Bisphenol A and phthalates in children with Chronic Kidney Disease. *Environ. Res.* **2018**, *167*, 575–582, doi:10.1016/j.envres.2018.08.006.
8. Kang, H.; Kim, S.; Lee, G.; Lee, I.; Lee, J.P.; Lee, J.; Park, H.; Moon, H.-B.; Park, J.; Kim, S.; et al. Urinary metabolites of dibutyl phthalate and benzophenone-3 are potential chemical risk factors of chronic kidney function markers among healthy women. *Environ. Int.* **2019**, *124*, 354–360, doi:10.1016/j.envint.2019.01.028.
9. Shen, Y.; Liu, T.; Shi, Y.; Zhuang, F.; Lu, J.; Zhu, Q.; Ding, F. Bisphenol A analogs in patients with chronic kidney disease and dialysis therapy. *Ecotoxicol. Environ. Saf.* **2019**, *185*, doi:10.1016/j.ecoenv.2019.109684.
10. Lee, I.; Park, J.Y.; Kim, S.; An, J.N.; Lee, J.; Park, H.; Jung, S.K.; Kim, S.Y.; Lee, J.P.; Choi, K. Association of exposure to phthalates and environmental phenolics with markers of kidney function: Korean National Environmental Health Survey (KoNEHS) 2015-2017. *Environ. Int.* **2020**, *143*, doi:10.1016/j.envint.2020.105877.
11. Jacobson, M.H.; Wu, Y.; Liu, M.; Attina, T.M.; Naidu, M.; Karthikraj, R.; Kannan, K.; Warady, B.A.; Furth, S.; Vento, S.; et al. Serially assessed bisphenol A and phthalate exposure and association with kidney function in children with chronic kidney disease in the US and Canada: A longitudinal cohort study. *PLOS Med.* **2020**, *17*, doi:10.1371/journal.pmed.1003384.
12. Kang, H.; Lee, J.P.; Choi, K. Exposure to phthalates and environmental phenols in association with chronic kidney disease (CKD) among the general US population participating in multi-cycle NHANES (2005-2016). *Sci. Total Environ.* **2021**, *791*, 148343, doi:10.1016/j.scitotenv.2021.148343.
